# Effect of four or more antenatal care visits on facility delivery and early postnatal care services utilization in Uganda: a propensity score matched analysis

**DOI:** 10.1186/s12884-021-04354-8

**Published:** 2022-01-03

**Authors:** Edson Mwebesa, Joseph Kagaayi, Anthony Ssebagereka, Mary Nakafeero, John M. Ssenkusu, David Guwatudde, Nazarius Mbona Tumwesigye

**Affiliations:** Makerere University School of Public Health, Kampala, Uganda

**Keywords:** Antenatal care, Facility-based delivery, Postnatal care, Propensity Score Matched Analysis, Uganda

## Abstract

**Introduction:**

Maternal mortality remains a global public health issue, more predominantly in developing countries, and is associated with poor maternal health services utilization. Antenatal care (ANC) visits are positively associated with facility delivery and postnatal care (PNC) utilization. However, ANC in itself may not lead to such association but due to differences that exist among users (women). The purpose of this study, therefore, is to examine the effect of four or more ANC visits on facility delivery and early PNC and also the effect of facility-based delivery on early PNC using Propensity Score Matched Analysis (PSMA).

**Methods:**

The present study utilized the 2016 Uganda Demographic and Health Survey (UDHS) dataset. Women aged 15 – 49 years who had given birth three years preceding the survey were considered for this study. Propensity score-matched analysis was used to analyze the effect of four or more ANC visits on facility delivery and early PNC and also the effect of facility-based delivery on early PNC.

**Results:**

The results revealed a significant and positive effect of four or more ANC visits on facility delivery [ATT (Average Treatment Effect of the Treated) = 0.118, 95% CI: 0.063 – 0.173] and early PNC [ATT = 0.099, 95% CI: 0.076 – 0.121]. It also found a positive and significant effect of facility-based delivery on early PNC [ATT = 0.518, 95% CI: 0.489 – 0.547].

**Conclusion:**

Policies geared towards the provision of four or more ANC visits are an effective intervention towards improved facility-based delivery and early PNC utilisation in Uganda.

## Introduction

Maternal mortality, defined as death of a mother due to complications from child birth or pregnancy, is still an important global public health problem [1], majorly in low income countries such as Sub-Saharan African countries [2] due to majorly poor maternal health services utilization (MHSU) [3]. Globally, approximately 0.3 million women and adolescent girls died in 2015 from pregnancy and childbirth-related complications and 2.6 million stillbirth babies occurred [4, 5] with 60% of stillbirth occurring during the antepartum period due to untreated infections, poor fetal growth and hypertension [6]. The Sustainable Development Goal (SDG) 3.1 aims at reducing maternal mortality ratio to less than 70 per 100,000 live births globally by 2030. However, based on recent trends, maternal mortality remains a huge challenge [7].

In 2015, Uganda was ranked among the top ten countries with the highest maternal mortality in the world, with a maternal mortality rate of 343 per 100 000; and number of maternal deaths of 57,000 mothers [4]. Stillbirth, maternal mortality and morbidity and other poor maternal health outcomes are high in Uganda and are associated with inadequate utilization of maternal health services, including inadequate utilization of antenatal care (none, incomplete and late ANC attendance), failure to deliver in health facilities, untimely postnatal checkups or no checkups at all [8–14].

The promise of early and full attendance of ANC visits is that, it would improve facility-based deliveries, postnatal care utilization and consequently improve maternal and child health [15, 16]. During pregnancy, ANC attendance plays an important role towards positive pregnancy outcomes because it is through these visits that screening and treatments of pregnancy complications such as preeclampsia, anemia, sexually transmitted infections, and non-communicable disease such as diabetes is done. Other services provided during this time include weight and height measure, tetanus immunization, provision of supplements such as folic acid, provision of information on behavioral modification and prevention and treatment of intermittent malaria [17–19]. Without proper management of pregnancy, adverse pregnancy outcomes such as low birth weight, preterm delivery, spontaneous abortion maternal and perinatal mortality and morbidity may result [18, 20].

Most studies have investigated factors affecting ANC, facility delivery, skilled birth attendance and postnatal care, and some studies have investigated how ANC affects neonatal and infant mortality, its association to low birth weight, stunting and underweight [21] and its relationship with facility-based delivery and perinatal survival [22]. Through the use of conventional logistic regression, positive associations between ANC attendance on facility-based delivery [23–30] and PNC utilization [31–37] have been observed. In addition, facility-based delivery has been associated with PNC utilization [38–42].

However, ANC in and of itself may not directly result in facility delivery and early PNC utilization, rather it may be due to individual differences in unknown factors that enable facility-based delivery and early PNC among mothers who utilize ANC [43]. For example, these mothers may be from wealthy households, educated and exposed to media. The use of propensity score matched analysis offers a better option compared to conventional logistic regression analyses in controlling for confounding that may exist in analyzing associations between ANC and facility delivery and early PNC utilization. The use of Propensity Score (PS) matches women who attended 4 + ANC visits (exposed) and those who attended less than 4 ANC visits (unexposed) with similar conditional probabilities of attending 4 + ANC visits hence reducing the bias that may persist when conventional logistic regression is used. This study applied PS matched analysis in examining whether a mother having had four or more ANC visits increases probability of facility-based delivery and early PNC utilization and also whether having facility-based delivery leads to increased probability of PNC utilization in Uganda. Four or more ANC visits were considered because, it is believed that having 4 + visits increases the likelihood of a pregnant woman receiving a full range of required maternal health interventions during pregnancy [44, 45] and by the time of data collection, the Uganda’s Ministry of Health equally recommended at least 4 or more ANCs for pregnant mothers. Studies that have examined the effect of ANC visits on health outcomes, specifically health facility delivery have used logistic regression models. Analyses using PSM to answer the same research question not only checks on consistency of previous results using another method but also reduces the bias in the intervention effect estimate.

Propensity score analysis (PSA) involves statistical methods for estimating treatment effects with observational data [46]. It offers an alternative approach for program evaluation in cases where randomized controlled trials are either infeasible, unethical or when researchers need to evaluate treatment effects from survey data. Associations between an outcome and given set of exposures may be biased due to unobservable individual characteristics in survey research. The use of propensity score matching (PSM) reduces such bias by matching women who attended 4 or more ANC visits (exposed) and those who attended less than 4 ANC visits (unexposed) with similar conditional probabilities to receive the treatment and is thus more preferred than traditional regression adjustments, such as logistic regression [43]. The PS is a balancing score that balances baseline characteristics between the exposed and unexposed groups based on survey data, therefore mimicking characteristics of randomized trials. [47–49]. It also helps create comparable balanced groups of respondents with respect to observed covariates and help minimize the influence of confounders such as age, education level, wealth index [50–53]. Propensity score matched analysis is used to estimate the average treatment effects of the treated (ATT) of a given covariate on outcome of interest [43, 54]. In this study, we assessed the effect of four or more ANC visits on facility-based delivery, the effect of four or more ANC visits on timing of PNC and the effect of facility-based delivery on timing of PNC using data drawn from Uganda Demographic and Health Survey of 2016.

## Methods

### Data Source and Study Population

This study used secondary data from the Uganda DHS of 2016. This is the most recent DHS survey conducted in over 20,000 households in all regions of Uganda carried out every after five years by Uganda Bureau of Statistics (UBOS). The study population comprised women of reproductive age (15–49 years) who had given birth three years preceding the survey. The Uganda DHS of 2016 used a two-stage cluster and stratified sampling technique to generate a nationally representative sample of women aged 15–49 years and men aged 15–59 years in the sampled households. Datails about the conduct of the survey can be found in Uganda Demographic and Health Survey key indicators report [55].

### Propensity Scores Analysis

In this study, we estimated the ATT of having 4 + ANC visits on facility delivery, and on early PNC check-up as the outcomes. We also estimated the ATT of facility delivery on early PNC check-up (having a PNC check-up after delivery within 48 h) as another outcome. A 1:1 ratio was used for propensity score matching [52, 56] and were constructed using individual-/household and community specific variables such as age, education level of the mother, and wealth index and type of place of residence. In this study, logistic regression was used as an estimation algorithm and radius and kernel as the matching algorithm from 0.05 to 0.08 tolerance level [47]. According to Rosenbaum and Rubin [57], a propensity score is the conditional probability of assignment to a particular treatment given a vector of observed covariates. It is generally expressed as follows: [Eq. 1]

$$\mathrm{p}\left(\mathrm{X}\right)=\mathrm{pr}(\mathrm{D}=1|\mathbf{X})$$

where $\mathrm{p}\left(\mathrm{X}\right)$ is the conditional probability of receiving a given exposure (4 + ANCs, facility delivery), $\mathrm{D}=(0, 1)$ is the exposure to appropriate covariate of interest and ${\varvec{X}}$ is a vector of covariates associated with ANC, facility delivery and early PNC check-up. The estimation of ATT follows a counterfactual framework and is expressed as: [Eq. 2]

$$\mathrm{ATT}=\mathrm{E}\left({\mathrm{Y}}_{1\mathrm{i}}|{\mathrm{D}}_{\mathrm{i}}=1\right)-\mathrm{E}({\mathrm{Y}}_{0\mathrm{i}}|{\mathrm{D}}_{\mathrm{i}}=1)$$

where $\mathrm{E}\left({\mathrm{Y}}_{1\mathrm{i}}|{\mathrm{D}}_{\mathrm{i}}=1\right)$ is the expected outcome of facility delivery and early PNC check-up (${\mathrm{Y}}_{1\mathrm{i}}$) if all exposed mothers received 4 + ANC visits (${\mathrm{D}}_{\mathrm{i}}=1$) and is also the expected outcome of early PNC check-up if all exposed mothers had a facility delivery. $\mathrm{E}({\mathrm{Y}}_{0\mathrm{i}}|{\mathrm{D}}_{\mathrm{i}}=1)$ is the expected outcome of facility delivery and early PNC check-up (${\mathrm{Y}}_{0\mathrm{i}}$) among mothers who received 4 + ANC visits had they not received 4 + ANC visits (unobserved). It also meant the expected outcome of early PNC check-up among mothers who received facility delivery if none of these mothers received facility delivery (unobserved) [43, 58–60].

The ATT is interpreted as the average difference in facility delivery and early PNC check-up that would be found if all treated women that have given birth preceding the survey received $\ge$ 4 ANC visits compared to the same women if they had not received 4 + ANC visits. It was also interpreted as the average difference in early PNC check-up that would be found if all treated women that had given birth in a health facility compared to the same women if they had not given birth in a health facility [59]. The steps that were followed are proposed by [59] in estimating treatment effects which are: 1) “Estimating propensity score”, 2)”stratifying and balancing propensity score”, and 3) “estimating causal effect”. Since it is impossible to observe the effects of treatment among women if they had received and not received 4 + ANC visits simultaneously, or if they have had facility-based delivery and not had facility-based delivery simultaneously [59], in this study, the counterfactual was constructed by matching women who received 4 + ANC visits with those who did not and women that had facility-based delivery and those that did not on a set of observable characteristics. Thus, a woman who did not receive 4 + ANC visits given that they had given birth three years preceding the survey served as a counterfactual case to those who received 4 + ANC visits. Also, women who had not given birth at a health facility preceding the survey served as a counterfactual case to those who did [43].

**Fig. 1:**
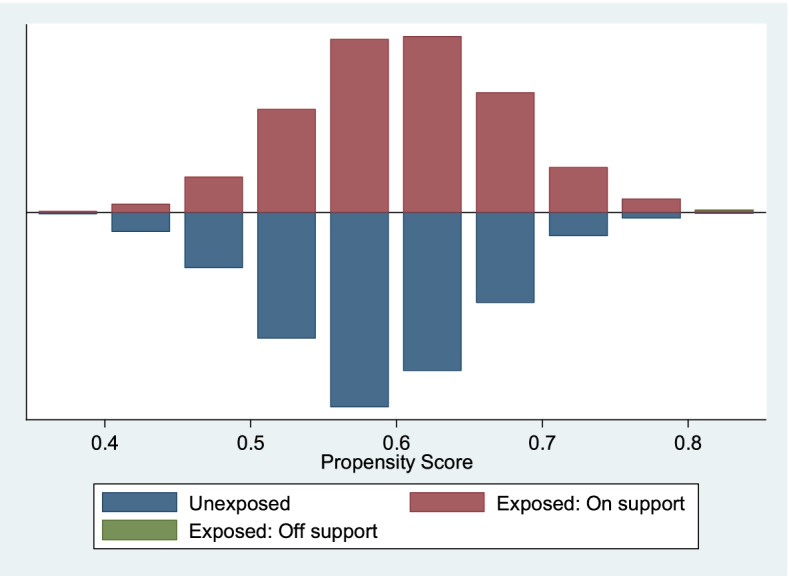
Propensity Scores across Four or more ANCs (1 = Exposed, 0 = Unexposed) by Facility Delivery. Source: UDHS Data 2016

In assessing the balance of propensity score across women who had four or more ANC visits (treatment) and those who had less than four ANC visits (comparison), and also for women who gave birth in a health facility (treatment) and those who did not (comparison), graphs of propensity scores across these groups were used. These graphs helped to determine whether there was an overlap in the range of propensity scores across groups (treatment and comparison groups) also called common support [61]. The graphs show that there is an overlap between propensity scores and there exist similar distribution (balance) between the treated and untreated groups. The Graphs are shown in Fig. 1 to Fig. 3.

## Results

After matching, a weighted sample of 7,903 women aged 15 – 49 years formed a sample in this study. This section presents the characteristics of women in this study, effect of 4 + ANC visits on facility-based delivery, effect of 4 + ANC visits on early PNC check-up, and the effect of facility-based delivery on early PNC check-up.

### Characteristics of Women Aged 15 – 49 Years

Most of the women who had given birth three years preceding the survey were young, aged 15 – 24 years with 3,140 (39.4%), had primary level of education 4,784 (60.5%), had some kind of work 6,494 8 (82.2%). Of these women, almost 4,723 (60%) perceived the distance to the health center as not a big problem, 2,686 (34%) used modern contraceptives. Most of the women had access to some form of media (newspapers, radio, or television) with 6,079 (77%) and had not had caesarean birth 7,315 (93%). Most women had a preceding birth interval of 2 to 5 years, 4,069 (66%) and were from households with low wealth index (poor and poorer) with 3,409 (43%). Most of the women were from rural areas 6,167 (78%) and from Eastern Uganda 2,175 (27.5%). The rest of the results are presented in Table 1

**Table 1:** Distribution of Women Aged 15–49 Years by Selected Background Characteristics, Using Data Derived from Uganda DHS 2016

Background Characteristics	Category	Count	Percent
Age groups (*n* = 7,903)	15 – 24	3,140	39.4
	25 – 29	1,965	24.9
	30 – 49	2,799	35.4
Mother’s level of education (*n* = 7,903)	No education	778	9.9
	Primary	4,784	60.5
	Secondary or higher	2,341	29.6
Employment Status (*n* = 7,903)	Not working	1,405	17.8
	Working	6,494	82.2
Distance to nearest health facility (*n* = 7,903)	Big problem	3,180	40.2
	Not a big problem	4,723	59.8
Modern contraceptive Use (*n* = 7,903)	Non user	5,217	66
	User	2,686	34
Mass media exposure (*n* = 7,903)	No	1,824	23.1
	Yes	6,079	76.9
Last birth a caesarean section (*n* = 7,878)	No	7,315	92.9
	Yes	563	7.1
Preceding birth interval (*n* = 6,151)	Short (< 24 months)	1,325	21.5
	Optimal (24–59 months)	4,069	66.1
	Long (60 months or more)	758	12.3
Household wealth index (*n* = 7,903)	Low	3,409	43.1
	Middle	1,475	18.7
	High	3,019	38.2
Type of place of residence (*n* = 7,903)	Urban	1,737	22
	Rural	6,167	78
Region (*n* = 7,903)	Central	2,166	27.4
	Eastern	2,175	27.5
	Northern	1,621	20.5
	Western	1,942	24.6

Note: The sample sizes stated above are weighted. Source: UDHS Data 2016.

**Fig. 2:**
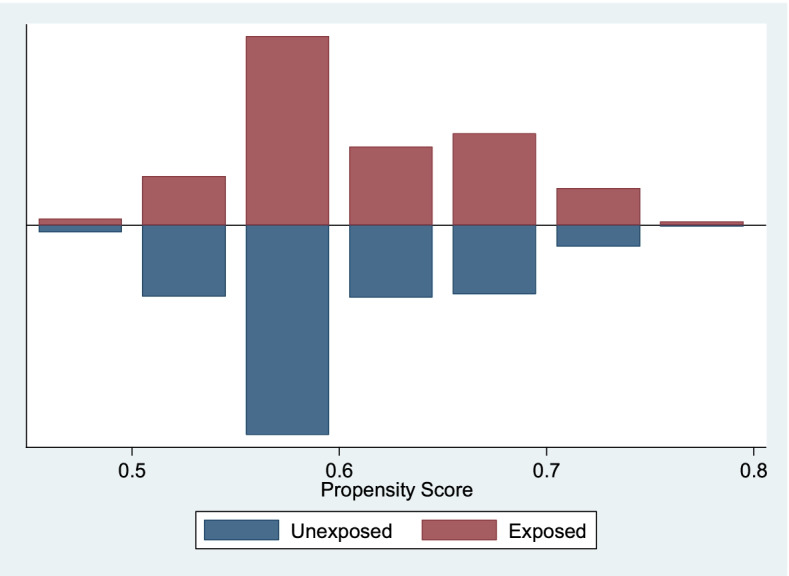
Propensity Scores across Four or more ANCs (1 = Exposed, 0 = Unexposed) by Early PNC Use. Source: UDHS Data 2016

**Fig. 3:**
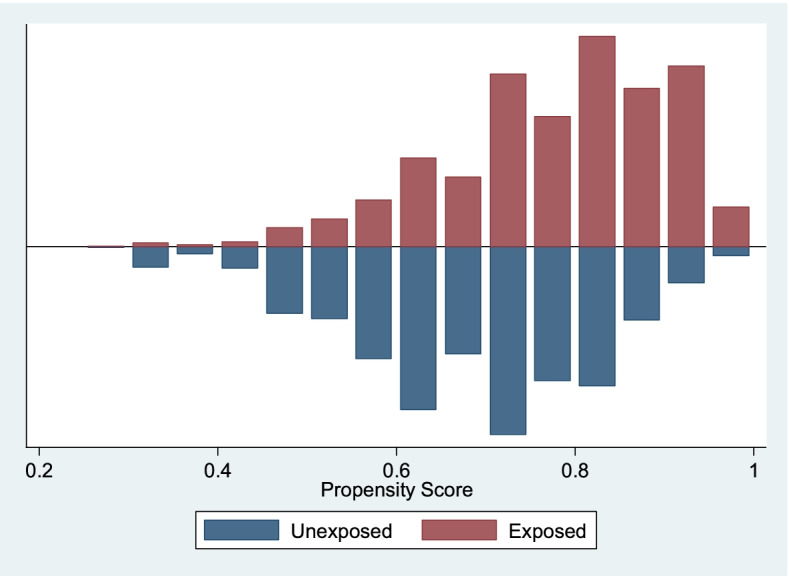
Propensity Scores across Facility Delivery (1 = Exposed, 0 = Unexposed) by Early PNC Use. Source: UDHS Data 2016

The propensity score graph evaluating the quality of matching among women who have had 4 + ANC visits (exposed) versus those who had not had 4 + ANC visits by facility-based delivery, shows that the groups are balanced and have, to a large extent similar distribution. The off support is negligible. This implies that the two groups can then be compared. See Fig. 1 above. The propensity score graph evaluating the quality of matching among women who have had 4 + ANC visits (exposed) versus those who had not had 4 + ANC visits by early PNC check-up, shows that the groups are balanced and have similar distribution. There was no off support. This implies that the two groups are matched. See Fig. 2 below. The propensity score graph evaluating the quality of matching among women who have had facility-based delivery (exposed) versus those who had not had a facility-based delivery (unexposed) by early PNC check-up, shows that the groups are balanced and have similar distribution. There was no off support. This implies that the two groups are matched. See Fig. 3 below.

The descriptive statistics obtained after matching on estimation of ATT on facility-based delivery and early PNC (EPNC) check-up reveal that the ATT (the difference among women who received 4 or more ANC visits and of the same women had they not had 4 or more ANC visits (counterfactual) on facility-based delivery and early PNC) is 0.118 ± 0.030 (almost 12%) on facility-based delivery and 0.099 ± 0.013 (almost 10%) on early PNC. The effect of facility-based delivery on early PNC was also investigated. The study found out that the ATT of facility-based delivery on early PNC was 0.518 ± 0.013 (52%). The results are presented in Table 2. The inferences about these results are shown in Table 3, where the standard errors were bootstrapped with 150 repetitions.

**Table 2:** Estimation of ATT of Four or More ANCs on Facility Delivery and Early PNC and of Facility Delivery on Early PNC

Variable	Sample	Exposed	Unexposed	Difference	SE	T-stat
***Estimation of ATT of Four or More ANCs on Facility Delivery and Early PNC***						
Facility Delivery	Unmatched	0.777	0.622	0.155	0.012	13.47
	ATT	0.777	0.659	0.118	0.03	3.87
Early PNC Use	Unmatched	0.587	0.474	0.113	0.013	8.6
	ATT	0.587	0.488	0.099	0.013	7.4
***Estimation of ATT of Facility Delivery on Early PNC***						
Facility Delivery	Unmatched	0.677	0.119	0.557	0.013	42.33
	ATT	0.676	0.158	0.518	0.013	40.74

Source: UDHS Data 2016.

**Table 3:** Quality of Matching and Average Treatment Effects on the Treated (ATT) of Four or More ANC Visits on Facility Delivery and Early PNC Use and of Facility Delivery on Early PNC Use

		Model Diagnostics					
		Pseudo R^2^	LR Chi2	*P * > chi2	Mean Bias	Median Bias	ATT [95% CI]
ANC (4 or more ANC visits) on Facility Delivery and EPNC							
Facility Delivery (*n* = 6,233)	Unmatched	0.019	163.04	0	8.7	8.3	
	Radius, Caliper (0.05)	0.002	22.74	0.09	2.4	1.2	0.118 [0.063, 0.173]^a^
Early PNC Use (*n* = 5,976)	Unmatched	0.011	84.48	0	8.6	7.7	
	Kernel, bwidth(0.08)	0.002	20.24	0.009	3.4	1.5	0.099 [0.076, 0.121]^a^
Facility Delivery on EPNC							
Early PNC Use (*n* = 5,976)	Unmatched	0.085	564.04	0	22.9	21.8	
	Kernel, bwidth(0.08)	0.002	29.84	0.001	3.1	1.7	0.518 [0.489, 0.547]^a^

Source: UDHS Data 2016. Note: Standard Errors were bootstrapped with 150 Repetitions, ^a^*p * <  *0.001*

After matching, the probability of facility-based delivery was 12% [ATT = 0.118; 95% CI = 0.063 – 0.173] higher among women who had 4 + ANCs compared to the same women had they not received 4 + ANC visits. This indicates that having a 4 + ANC visits increases the chances of having a facility-based delivery by 12% compared to when they have not had 4 + ANC visits. Regarding the effect of 4 + ANC visits on early PNC check-up, it was found out that the probability of early PNC check-up was 10% [ATT = 0.099; 95% CI = 0.076 – 0.121] higher among women who had 4 + ANCs compared to the same women had they not received 4 + ANC visits. This implies that having had 4 + ANC visits increases the chances of early PNC check-up by 10%. On the effect of facility-based delivery of early PNC check-up, the results revealed that the probability of early PNC was 52% [ATT = 0.518; 95% CI = 0.489 – 0.547] higher among women who had facility-based delivery compared to the same women had they not had facility-based delivery. This implies that having had a facility delivery increases the chances of early PNC check-up by 52%. Over all, these results reveal that ANC visits (four or more ANC visits) significantly affect the probability of facility-based delivery and early PNC. They also show that facility-based delivery significantly affects the probability of early PNC. Results are presented in Table 3.

## Discussion

We found that ANC attendance of 4 + visits was associated with a 12% higher probability of health facility-based delivery compared to the same women had they not attended 4 + ANC visits. We also found out that ANC attendance of 4 + visits was associated with a 10% higher probability of early PNC check-up among women compared to the same women had that not attended 4 + ANC visits. The study also revealed that having a health facility-based delivery was associated with 52% higher probability of early PNC check-up compared to the same women had they not had had facility-based delivery.

Literature shows that ANC attendance during pregnancy is positively associated with facility-based delivery [24, 26, 28, 29] and PNC utilization [31, 32, 37]. It also shows that facility-based delivery is positively associated with PNC utilization [41, 42], based on conventional regression models. The present study revealed a significant and positive effect of 4 + ANC visits on facility-based delivery and EPNC utilisation, and facility-based delivery on early PNC utilisation after matching exposed and unexposed women on observable and significant characteristics within 2016 UDHS dataset.

The results align with previous studies which highlighted a positive association between appropriate ANC attendance and facility delivery and PNC utilisation [24, 26, 28, 29, 31, 32, 34, 37] and with those carried out in Uganda [11, 13, 62, 63]. Regarding the effect of 4 + ANC visits on facility-based delivery, our results agree with similar studies linking ANC with facility-based delivery in Bangladesh and India that used propensity score matched analysis [43, 58]. This is likely due to the fact that women who attend ANC receive maternal education and are often referred to health facilities for delivery [43].

The study further observed that ANC attendance affects early PNC utilisation and also that facility-based delivery affected early PNC utilisation. Studies using propensity score matched analysis investigating these effects could not be found in literature. Overall, the findings of this study confirm the belief that ANC attendance improves the likelihood of facility-based delivery and PNC utilisation and also that facility-based delivery improves the probability of early PNC use.

However, the results from this study are based on observational data to infer causality or causal relationship between; 4 + ANC visits and facility-based delivery, 4 + ANC visits and early PNC utilisation and facility-based delivery and early PNC utilisation. Even though propensity score matching removes bias based on observable woman characteristics, bias due to unobservable confounders is not accounted for leading to overestimated effects of ANC visits on facility-based delivery and early PNC utilisation and facility-based delivery on early PNC utilisation [43]. However, the use of propensity scores provides a better method for assessing interventions where the use of controlled randomized trial is impossible or inappropriate. It matches the treated with controls based on observable confounders which leads to better estimates of treatment effect. It ensures covariate balance across groups leading to unbiased estimates through the use of observational data.

## Conclusions and Recommendations

The results from propensity score matched analysis illustrate a significant and positive relationship between; 4 + ANC visits and facility-based delivery, 4 + ANC visits and early postnatal care utilisation, and facility-based delivery and early postnatal utilisation among mothers in Uganda. The implementation of policies towards provision of ANC services (at least four ANC visits) plays as an effective intervention to increase facility-based delivery and ultimately early postnatal utilisation in Uganda.

## Data Availability

The datasets generated and/or analyzed during the current study are publicly available in the Demographic Health Survey repository, https://dhsprogram.com/data/available-datasets.cfm.
